# The Onset of Rapid-Guessing Behavior Over the Course of Testing Time: A Matter of Motivation and Cognitive Resources

**DOI:** 10.3389/fpsyg.2019.01533

**Published:** 2019-07-23

**Authors:** Marlit Annalena Lindner, Oliver Lüdtke, Gabriel Nagy

**Affiliations:** ^1^IPN - Leibniz Institute for Science and Mathematics Education, Kiel, Germany; ^2^Centre for International Student Assessment, Munich, Germany

**Keywords:** rapid-guessing behavior, motivation, test-taking effort, item position effect, low-stakes assessment, large-scale assessment (LSA), latent class analysis

## Abstract

Digital tests make it possible to identify student effort by means of response times, specifically, unrealistically fast responses that are defined as rapid-guessing behavior (RGB). In this study, we used latent class and growth curve models to examine (1) how student characteristics (i.e., gender, school type, general cognitive abilities, and working-memory capacity) are related to the onset point of RGB and its development over the course of a test session (i.e., item positions). Further, we examined (2) the extent to which repeated ratings of task enjoyment (i.e., intercept and slope parameters) are related to the onset and the development of RGB over the course of the test. For this purpose, we analyzed data from *N* = 401 students from fifth and sixth grades in Germany (*n* = 247 academic track; *n* = 154 non-academic track). All participants solved 36 science items under low-stakes conditions and rated their current task enjoyment after each science item, constituting a micro-longitudinal design that allowed students' motivational state to be tracked over the entire test session. In addition, they worked on tests that assessed their general cognitive abilities and working-memory capacity. The results show that students' gender was not significantly related to RGB but that students' school type (which is known to be closely related to academic abilities in the German school system), general cognitive abilities, and their working-memory capacity were significant predictors of an early RGB onset and a stronger RGB increase across testing time. Students' initial rating of task enjoyment was associated with RGB, but only a decline in students' task enjoyment was predictive of earlier RGB onset. Overall, non-academic-school attendance was the most powerful predictor of RGB, together with students' working-memory capacity. The present findings add to the concern that there is an unfortunate relation between students' test-effort investment and their academic and general cognitive abilities. This challenges basic assumptions about motivation-filtering procedures and may threaten a valid interpretation of results from large-scale testing programs that rely on school-type comparisons.

## Introduction

Computer-based assessments are being implemented more and more in educational institutions and large-scale testing programs. This digitalization of tests makes response-time measures (i.e., time on task; e.g., Goldhammer et al., 2014) and log files (e.g., Greiff et al., 2015) easily available. This opens new paths to more objective and also deeper insights into students' test-taking behavior (e.g., Wise and Kong, 2005; Goldhammer et al., 2014; Finn, 2015), for example, by detecting rapid-guessing behavior (RGB). The term RGB basically means that a test-taker provides a response to an item in just a few seconds after the item has been presented. Given that it is highly implausible that students truthfully work on a given task in such a short time frame, RGB is interpreted as a reflection of non-effort (Wise and Kong, 2005; Goldhammer et al., 2016; Wise, 2017). Even though RGB has recently been subject to valuable investigations that shed more light on the nature of this undesirable test-taking behavior, the psychological determinants that are related to RGB in low-stakes assessment have not yet been sufficiently examined.

The present study takes a closer look at the correlates of RGB, placing a specific focus on students' individual probability of showing an early RGB onset over the course of testing time. Specifically, we aimed to investigate the role of two main explanatory psychological characteristics at a student level that are considered to be related to low test-taking effort, namely, a lack of motivational and cognitive resources.

### Motivation and Test-Taking Behavior

Educational assessment is essential for the evaluation of learning outcomes and the determination of the proficiency levels of test takers in diverse contexts. Unfortunately, test takers are not always fully motivated to engage in solving test items, especially in low-stakes settings (e.g., Wise and DeMars, 2005, 2010; Wise, 2006; Finn, 2015). Low-stakes means that the test scores have no formal consequences at a student level (e.g., grades, graduation), although aggregated test scores often have major consequences at an institutional or governmental level (e.g., program funding, educational reforms). A high level of effort invested by students when working on a test is considered a prerequisite for a reliable and valid interpretation of achievement levels (Cronbach, 1960; Messick, 1989; Baumert and Demmrich, 2001; Goldhammer et al., 2016). If the problem of low test-taking effort is not treated, for example by statistical correction procedures, students' proficiency may be underestimated, which may lead—in turn—to biased conclusions (see e.g., Wise and DeMars, 2005; Wise et al., 2006b; Nagy et al., 2018b).

Low test-taking motivation in low-stakes assessments is often explained by *Expectancy-Value Models* (e.g., Eccles et al., 1983; Wigfield and Eccles, 2000; Eccles and Wigfield, 2002), which assume that achievement motivation for a given task (e.g., taking a test) is a function of (1) *expectancy* (i.e., students' expectation of success in solving the test items) and (2) *value* (i.e., the perceived importance and usefulness of the test). The expectancy component is determined by both students' abilities and task demands and is, for example, low when test items are too difficult for a student. The value component is considered to be more complex: Eccles and Wigfield (2002) distinguish between four value components, namely, (a) attainment value (e.g., task importance), (b) intrinsic value (e.g., task enjoyment), (c) utility value (e.g., relevance for future goals), and (d) perceived costs (e.g., effort). It can be assumed that all four of these value aspects and, thus, also the overall value component are rather low in low-stakes assessments. This is because, at least for some test takers, the lack of personal consequences and a lack of intrinsic value in taking the test may be in conflict with the effort that is required to successfully solve the items. This is especially true for students with lower competence levels (i.e., low expectancy) who need to invest more effort to successfully work on a test. Accordingly, based on expectancy-value models, achievement motivation can be expected to be lower in low-performing students than in high-performing students.

Lower levels of student motivation become a serious problem when they result in low test effort, which can be defined as a lack of mental work that is put into responding to test items (Wise and DeMars, 2005, 2010; Finn, 2015). Analyzing data sets that include such invalid responses threatens the interpretation of the test scores obtained because construct-irrelevant variance is introduced (Haladyna and Downing, 2004; Nagy et al., 2018a) and psychometric properties are deformed (see e.g., Rios et al., 2017). This issue is often addressed by motivation-filtering procedures (see e.g., Finn, 2015, for a review): As one option, filtering can be based upon self-report questionnaires that aim to assess students' global test-taking motivation (e.g., Student Opinion Scale; Thelk et al., 2009). Such measures are convenient in any type of assessment (including paper-pencil tests), but self-reports are more vulnerable to measurement errors and social desirability (Swerdzewski et al., 2011). As a second option, measuring response times in computer-based assessments provides unobtrusive, more objective insights into students' actual test-taking behavior (e.g., Wise and Kong, 2005; Greiff et al., 2015), while this measure does not disturb or influence students during their taking of the test. Typical sources of measurement error can thus be minimized when referring to students' response behavior as an indicator of effort (or non-effort).

### Identifying Rapid-Guessing Behavior

The identification of RGB has proven useful for detecting test takers who do not exert their maximum effort in a test (e.g., Wise, 2006, 2017; Wise et al., 2006b; Finn, 2015). RGB is operationalized by unrealistically low response times that would not even allow the item content to be read and understood and especially would not allow an effortful response; any trial that is not identified as RGB is considered solution behavior, resulting in a dichotomous measure of RGB. However, it is noteworthy that responses that are categorized as solution behavior do not necessarily reflect effortful item solving (for a discussion see e.g., Finn, 2015; Wise, 2017). The main advantage of identifying RGB is that it can be measured for each student and each item. This means that all single trials (i.e., person × item interaction) can be classified as either RGB or solution behavior (see e.g., Wise and Kong, 2005), which makes it possible, for example, to trace the development of non-effort over the course of the test.

However, a reasonable response time threshold needs to be determined to separate (non-effortful) RGB responses from (probably effortful) solution behavior. In doing so, false-positive and false-negative classifications need to be avoided. Various approaches have been discussed (e.g., Wise and Kong, 2005; Wise, 2006; Kong et al., 2007; Wise and Ma, 2012; Lee and Jia, 2014; Finn, 2015; Goldhammer et al., 2016). Defining one constant threshold for every item (e.g., 3 s) is a basic option to determine RGB. However, item-specific, normative thresholds that vary as a function of the mean response time per item (i.e., a certain percentage of the item mean is used to separate RGB from solution behavior; see e.g., Wise and Ma, 2012; Lee and Jia, 2014) or item characteristics (Wise and Kong, 2005; Wise, 2006) often yield a more valid classification of RGB and solution behavior. This is because item attributes can substantially impact the meaning and interpretation of (short) response times. Nonetheless, the different approaches can be helpful in handling different types of data sets (see e.g., Wise, 2017). Thresholds further need to be cross-validated by a combination of different criteria for every test (see e.g., Goldhammer et al., 2016; Wise and Gao, 2017). For example, the accuracy of responses classified as RGB should equal the a priori guessing probability per item, thresholds should be validated by the visual inspection of response time distributions, and 10-s thresholds should not to be exceeded. However, smaller threshold changes do not have a substantial impact on further analyses, suggesting that RGB can be classified with a high reliability—more or less independent of the specific method applied (Kong et al., 2007).

In conclusion, from a pragmatic perspective, RGB can serve as a useful indicator of test-takers' non-effort in motivation-filtering procedures. However, it is also important to gain a better understanding of RGB at a theoretical level and from a psychological point of view.

### Theories and Correlates of Rapid-Guessing Behavior

Expectancy-value models help to predict achievement motivation in low-stakes tests. Related assumptions that are more specific to the assessment context and the explanation of RGB have been proposed by Wise and Smith (2011) in the *Demands-Capacity Model* (DCM; see also Wise, 2017). The core of the DCM is the assumption that the tendency of a test taker to engage in RGB is a function of the current fit of (1) the resource demands of the presented items, and (2) the effort capacity of the student. Resource demands are defined as aspects of an item that determine how difficult or mentally taxing it is, such as higher reading demands or complex information. On the other side, test-takers are assumed to have a certain effort capacity that they can invest in solving an item at a specific moment. The DCM is still vague regarding the factors that determine the current status of effort capacity, as the authors propose that many factors have an influence, namely, “test stakes, time pressure, fatigue from answering earlier items, how interesting earlier items were, or a desire to please teachers or parents” (Wise, 2017, p. 53). The DCM further assumes that students compare the current item demands with their current effort capacity. They decide to engage in solution behavior for a given item when their effort capacity is sufficient or, otherwise, to engage in RGB. This explains that test-takers change their response pattern in reaction to different items, as both item demands and effort capacity can easily fluctuate across a test session. Even though RGB is commonly understood as an indicator of a lack of motivation (see e.g., Finn, 2015), building on the DCM, we assume that students might also refuse to work on an item when they lack basic cognitive resources (i.e., as a facet of a lower effort capacity).

Evidence supporting the DCM comes from studies that have investigated correlates of RGB. There are two typical levels of aggregation: the person and the item level. Regarding the student level, the measure of response time effort (RTE^1^), as introduced by Wise and Kong (2005), is determined as the proportion of solution behavior related to all presented items in a test and provides information concerning the overall level of invested effort per student. The correlations of RTE and person characteristics can provide information concerning factors that go along with higher or lower levels of test-taking effort, respectively. The item-specific counterpart, introduced by Wise (2006), is response time fidelity (RTF). It represents the effort invested in a specific item across all test-takers, namely, the proportion of effortful responses to that item. Thus, RTF is a useful parameter to investigate correlates of effort based on item characteristics. It is also possible to model students' responses by more complex linear or generalized mixed-effects models (e.g., Wise et al., 2009) to jointly investigate student and item characteristics and their connections to RGB.

Building on RTE and RTF and using multilevel approaches, research has shown that higher RGB prevalence at a student level (i.e., RTE) is, for example, often associated with lower academic abilities (e.g., Wise et al., 2009; Lee and Jia, 2014; Goldhammer et al., 2016; Wise and Gao, 2017), male gender (e.g., DeMars et al., 2013; Goldhammer et al., 2016), personality traits, such as lower conscientiousness and agreeableness or higher neuroticism (e.g., DeMars et al., 2013; Barry and Finney, 2016; Lu et al., 2018), and cultural background characteristics (e.g., Goldhammer et al., 2016). However, the findings are not consistent across studies. Especially the relation of test effort and academic ability levels needs to be discussed and investigated more as the results are mixed and of high practical importance (see e.g., Wise and DeMars, 2005; Wise and Kong, 2005; Wise et al., 2006b, 2009; Kong et al., 2007; Lee and Jia, 2014; Goldhammer et al., 2016; Wise and Gao, 2017). Overall, previous findings align with the DCM as they suggest that academic and motivational resources as well as sociocultural aspects play a role in test-takers' effort capacity, which is assumed to be responsible for their decisions to show solution behavior or to engage in RGB instead.

Again in line with DCM assumptions, there is evidence that item characteristics (i.e., item demands) influence students' tendency to engage in RGB. Especially surface characteristics, such as shorter texts and the presence of pictures have been shown to be related to lower RGB rates (Wise et al., 2009; Lindner et al., 2017a). However, deep item characteristics that are not easily traceable at first sight, such as item difficulty or the content area of an item did not have a significant impact on RGB rates, as shown by Wise et al. (2009). From a logical point of view, this is not surprising because the short time frame in which students look at an item before they engage in RGB is not long enough to analyze deeper item characteristics. Thus, the item appearance seems to be more important for the perception of item demands and the decision to engage in RGB or not.

Furthermore, the circumstances of the test situation have been connected to test-taking effort and RGB rates. For example, although different seasons or weekdays did not influence students' test-taking effort, a later testing time in a day (e.g., testing in the afternoon) was linked to lower RTE measures (i.e., more RGB; Wise et al., 2010). This suggests that physical and/or mental fatigue plays a role in reduced test-taking effort (Lindner et al., 2018), which may also explain why the most important predictor of RGB is the elapsed testing time (see e.g., Wise et al., 2009). There is compelling evidence across studies that items presented in later positions in a test are typically solved with lower accuracy (item position effect; e.g., List et al., 2017; Weirich et al., 2017; Nagy et al., 2018a), less motivational effort (e.g., Barry and Finney, 2016; Penk and Richter, 2017) and are substantially more prone to RGB (e.g., Wise et al., 2009; Setzer et al., 2013;Goldhammer et al., 2016).

Consequentially, because test-item demands change neither with day times nor with the test duration, the existing findings indicate that the reported increase of RGB over the course of testing time is mostly related to changes at the level of test takers' resources. Overall, there is reason to assume that both motivational and cognitive capacities become exhausted over the course of testing time due to the effort that has already been invested in solving previous items. Specifically, students need to build a new situational mental model for every single item and cognitively switch between tasks and solution strategies in a short time frame (Lindner et al., 2017a). Such operations are demanding and require working-memory capacity (i.e., executive attention; Engle, 2002) and self-control (Lindner et al., 2017a). Following Inzlicht et al. (2014), investing self-control to focus attention on cognitive tasks becomes more and more aversive over time, leading to a motivational disengagement from effortful tasks while attentional disruptions increase. This is also presumed to go along with a negative influence on students' affect over the course of a test session, which may cause a reduction in motivational effort (e.g., Ackerman and Kanfer, 2009; Ackerman et al., 2010; Inzlicht et al., 2014). As a consequence, individuals' performance typically decreases over the course of the test (e.g., Penk and Richter, 2017;Nagy et al., 2018b).

In this study, based on the DCM, we assumed that increasing exhaustion and negative emotions would be more pronounced for students who have lower cognitive capacities (i.e., academic abilities, general cognitive abilities, and working-memory capacity) and lower motivational capacities (i.e., low task enjoyment). Thus, we expected that students with lower cognitive and motivational resources suffer from an earlier depletion of their effort capacity and, thus, start to engage in RGB at an earlier point in the testing time.

### The Present Research

Although different studies have investigated the correlates of RGB, they mainly considered the frequencies or proportions of RGB (i.e., RTE or RTF; e.g., Wise and Kong, 2005; Wise, 2006) and, to the best of our knowledge, no studies have yet focused on correlates for the RGB onset in a test session. Furthermore, the question remains open of whether a lower level of test motivation at the beginning of the test and a (faster) loss of motivation over the course of the test session are associated with an earlier RGB onset. The present study aimed to answer these questions by investigating the measures of student characteristics (i.e., gender, school type, general cognitive abilities, and working-memory capacity) as well as data from a micro-longitudinal design with 36 repeated ratings of students' task enjoyment over the course of testing. Our main goal was to investigate the relations of these cognitive and motivational measures to students' individual risk of early RGB onset during a test, in order to enhance the theoretical understanding of the RGB phenomenon.

Parts of the underlying data set have been previously published with a much different focus on the effects of representational pictures in testing (see Lindner et al., 2017b). RGB was one of three dependent variables in the investigation of the effects of pictures as an item design characteristic. We do not report the respective findings in this study but, rather, directly build on the prior insights regarding students' RGB development across time, which we summarize here very briefly. In line with the literature (e.g., Goldhammer et al., 2016), the data showed a substantial RGB increase over the course of the test session, indicated by a significant main effect of item position (see Lindner et al., 2017b). However, this increase was substantially smaller in items that contained a representational picture (significant main effect picture). There was no significant interaction between the factors picture and item position. Pictures mainly induced a shift in RGB frequency. Both text-only and text-picture items were subject to an increase in RGB across time, but the probability of RGB was smaller throughout the test for items that contained a picture. In the current analyses, we took the systematic variation of picture presence as a control factor into account, but did not specifically investigate this characteristic.

In line with the literature, we assumed in the present research that RGB is a type of behavior that, similar to other phenomena in the testing context (e.g., item position effects, performance decline; e.g., Hartig and Buchholz, 2012; Debeer et al., 2014; Jin and Wang, 2014; List et al., 2017; Weirich et al., 2017; Wise and Gao, 2017; Nagy et al., 2018b), has a high probability of being maintained (at a student level) over the course of a test session, once it has begun. This means that once individuals engage in RGB, they have a high probability of showing this behavior in the subsequent items of the test. This assumption is also in line with insights from raw data of individuals' RGB development as well as with the DCM (Wise, 2017), according to which a depletion of students' effort capacity across time goes along with a higher probability of engaging in RGB. This hypothesis also formed the base of our attempt to model the data in a latent class approach to investigate the correlates of students' RGB onset, which will be explained in detail in the Methods section. Specifically, drawing on the empirical and theoretical background in the field as outlined above, we formulated the following hypotheses:

*Hypothesis 1*: We expected to find a higher probability of earlier RGB onset in (a) male students, (b) students from non-academic-track schools, (c) students with lower cognitive resources in terms of general cognitive abilities, and (d) students with lower cognitive resources in terms of working-memory capacity.
*Hypothesis 2:* We expected that both the initial level of students' task enjoyment and its (negative) development over the course of testing would be predictive of RGB. Specifically, we expected that both (a) lower initial enjoyment ratings (intercept) and (b) a stronger decrease (slope) would be associated with the RGB variable and predict earlier RGB onset.

## Methods

As mentioned above, the current data set has been subject to investigations before. To avoid unnecessary repetition, we only report the measures that are relevant for the present analyses. Please consult the report by Lindner et al. (2017b) for further details.

### Sample, Material, and Study Design

The analyzed sample comprised *N* = 401 students in the fifth and sixth grades in northern Germany (53.4% female, 51.4% fifth grade, *M*_age_ = 10.74, *SD*_age_ = 0.76; *n* = 247 academic track [i.e., Gymnasium]; *n* = 154 non-academic track [i.e., regional school]) who took a computerized science test in an experimental classroom setting. Students were informed that their individual participation was completely voluntary and that they would not face any negative consequences if they did not participate or if they canceled their participation. Thus, all students were fully aware of the low-stakes testing environment, but they were also informed about the relevance of investing good effort to ensure reliable research results.

The scientific literacy test was constructed based on the science framework and items of the Trends in International Mathematics and Science Study (TIMSS; see e.g., Mullis et al., 2009; International Association for the Evaluation of Educational Achievement [IEA], 2013), which assess students' basic science achievement. The 36 items confronted students with realistic situations, forcing them to apply their declarative science knowledge from biology, physics, and chemistry to everyday phenomena and problems. It was essential that the students correctly understood the situation in the item stem for them to be able to solve the problem correctly. The items had a mean word count of *M* = 74.9 words (*SD*_words_ = 24.2). All items were presented in a multiple-choice format with a short item stem, a separate one-sentence question, and four answer options (one correct option). The items were randomly assigned to one of three test blocks (12 items per block), which were presented either with or without representational pictures (i.e., experimental manipulation of test items), resulting in six booklet constellations. A randomization check confirmed that the item difficulty did not differ between the blocks, *F*_(2, 33)_ = 0.05; *p* = 0.95; η^2^ = 0.003. The systematic variation of presenting a representational picture (or not) in the items was balanced across booklets and realized in a within-subject multi-matrix design. To investigate RGB over the course of the test (i.e., in different item positions), items were presented in a random order within test blocks to avoid presenting certain items in certain positions. The six booklets were randomly assigned to the students and equally distributed in the sample (including school types). The marginal EAP/PV reliability of the science test was estimated as *Rel*. = 0.83.

### Measures

#### Background Variables

We used a short questionnaire to assess background information, such as students' age, gender, grade level (fifth vs. sixth grade) and the attended school type (academic and non-academic track).

#### General Cognitive Abilities

The subtest N2 (Figural Analogies; adjusted according to students' grade level; α = 0.93/0.89) of the Kognitiver Fähigkeitstest (KFT) 4 – 12 + R (Heller and Perleth, 2000) was applied to measure spatial reasoning skills as an indicator of students' general cognitive abilities and resources. The subscale consists of 25 items, each of which presents students with one pair of meaningfully related figures and another single figure, for which the appropriate counterpart has to be selected from five answer options in order to create a similar pair of related figures.

#### Working-Memory Capacity

A self-programmed, computerized version of a reversed digit span test (see e.g., HAWIK-IV; Petermann and Petermann, 2010) served as an indicator of students' working-memory capacity. Students listened through headphones to an increasing number of digits (i.e., two up to eight) that were read out at a slow pace by a male voice. During the digit presentation, the keyboard was locked. After hearing each row (e.g., 3–5–8–7), students were asked to type the digit row in reverse order (e.g., 7,853) into a box that appeared on the screen. After logging in the response, the screen went white and the next digit row followed. The test contained 14 trials. The sum of correct answers determined the test score. Reliability was just sufficient (α = 0.64).

#### Task Enjoyment Ratings

As an indicator of students' current motivational level, we repeatedly measured students' task enjoyment while working on the items. We did so with a one-item measure (see Lindner et al., 2016), asking students how much fun they had solving the current item (i.e., “Working on this item was fun for me”). We assumed that lower enjoyment ratings would indicate lower motivational resources.

#### Rapid-Guessing Behavior

Students' response time was measured per item (in seconds), which served as the base for classifying RGB trials. Extreme response times two standard deviations (*SD*) above the item mean (0.3% of the data) were trimmed by replacing them with the value of two *SD* above the item mean (e.g., Goldhammer et al., 2014) to prevent bias in the means. Afterwards, the mean time on task for each item served as a base for setting RGB thresholds, following the normative threshold (NT) method proposed by Wise and Ma (2012). Using this method, item-specific threshold percentages can be defined, which means that response times shorter than, for example, 10%, 15%, or 20% of the average solution time of an item are classified as rapid guesses. To achieve a balance between identifying as many non-effortful responses as possible and avoiding the classification of effortful responses as RGB (e.g., Wise and Kong, 2005; Lee and Jia, 2014), we used a mixed approach to evaluate potential thresholds by different validation methods (i.e., absolute thresholds, visual inspection and guessing probability in RGB trials; e.g., Goldhammer et al., 2016; see also section Motivation and Test-Taking Behavior). Taking all validation criteria into account (for a detailed evaluation, see Lindner et al., 2017b), the NT15 criterion turned out to deliver the best fit and was thus used for the RGB definition. This resulted in an average item-specific threshold of *M* = 5.6 s (*SD* = 1.4).

#### Apparatus and Procedure

Experienced test administrators conducted the study at schools during lesson time. All sessions were attended by a teacher and lasted up to 90 minutes. A laptop, headphones, and a mouse were prepared for each student. The science items were presented on 28 identical Lenovo® laptops, using the software flexSURVEY 2.0 (Hartenstein, 2012). Students answered a short background questionnaire, worked on the KFT, and took the working-memory test. Afterwards, they worked on the science test. It was ensured that students knew that they would not be able to return to an earlier question after choosing an answer and that they always needed to provide a response in order to progress to the next item. Following each item, students rated their item-solving valence. Providing an answer automatically forwarded the student to the next task. Students were repeatedly encouraged to take all the time they needed to solve each item but to work in a focused way through the test. This was done to ensure that the science test was worked on as a power test. There was no time limit for completing the test. Responses, response times (per item), and the item presentation sequence (i.e., item positions) were recorded in a log file for each student.

#### Data Analyses

RGB is a low-frequency behavior that is not exhibited by each student. As such, statistical modeling approaches for RGB should divide the total sample into at least two groups (or latent classes): One class that does not show RGB at all, and a second class of respondents who show at least some RGB responses. Within the class of individuals showing some RGB, the representation of the distribution of RGB can be challenging, especially in samples of modest size.

As a solution to this problem, we modeled RGB by means of a categorical latent variable (i.e., a latent class analysis; LCA). Our LCA model distinguished between latent classes that showed no RGB at all (i.e., no-RGB class), and three other classes that differed in the onset points of RGB (i.e., early, intermediate, and late onset points). In addition, we assumed the existence of a latent class consisting of students who had a rather low but constant probability of RGB at any point in the test (i.e., constantly low RGB class). To achieve this goal, we modeled the logits of the probability of RGB indicators *y*_*ip*_ [*y*_*ip*_ = 1 if individual *i* (*i* = 1, 2,…, *N*) showed RGB in position *p* (*p* = 1, 2, …, 36), and *y*_*ip*_ = 0 otherwise] conditional on class membership *C*_*i*_ = *k* (*k* = 1, 2, 3, 4, 5):

(1) $$\begin{array}{l} \text{logit}\left[P\left(y_{ip}=1|C_{i}=k\right)\;\right]\\ =\gamma_{k}w_{ip}+\tau_{0k}+\frac{\theta_{k}}{1+\exp\left[-\alpha_{k}\left(\beta_{k}-p\right)\right]} \end{array}$$

In Equation (1), *w*_*ip*_ is a variable indicating whether the item presented in position *p* to individual *i* is a text-only (*w*_*ip*_ = 0) or a text-picture item (*w*_*ip*_ = 1), and γ_*k*_ is a logistic regression weight accounting for the fact that text-picture items are less likely to be associated with RGB (see Wise et al., 2009; Lindner et al., 2017b). The parameter γ_*k*_ was specified to be invariant across classes reflecting RGB (i.e., *C* = 1–4), but was constrained to zero in the no-RGB class (*C* = 5). The last two terms on the right-hand side of Equation (1) capture the development of RGB across item positions. τ_0*k*_ is a lower asymptote parameter, and θ_*k*_ describes the upper asymptote of the probability of RGB in class *C* = *k*. The parameter α_*k*_ (α_*k*_ ≥ 0) reflects the rate of change in RGB probabilities, whereas β_*k*_ stands for the position in which the inflection point of the logistic function occurs in class *C* = *k*.

In order to provide an interpretable solution, the LCA parameters of Equation (1) were subjected to further constraints. The first three classes (*C* ≤ 3) were specified to reflect students with different onset points of RGB (parameters β_*k*_). Here, we specified the β_*k*_ parameters to be ordered (i.e., β_1_ < β_2_ < β_3_) and equally spaced, and the lower and upper asymptotes, τ_0*k*_ and θ_*k*_, to be equal across these three classes. In order to provide an interpretable asymptote parameter, we constrained the rate-of-change parameter α_*k*_ in such a way that the RGB probability in *p* = 1 (i.e., first item position) in the late-RGB-onset class (*C* = 3) solely reflected the lower asymptote τ_0*k*_. To this end, we constrained the last term of Equation (1) to be very close to zero in *p* = 1 by imposing the constraint $\alpha_{k}=\frac{logit\left(0.001\right)}{\left(\beta_{3}-1\right)}$. The constantly low RGB class (*C* = 4) was assumed to have the same τ_0*k*_ and α_*k*_ parameters as the classes *C* = 1 to 3, but θ_4_ was allowed to take a different value. In this class, β_4_ was set to be equal to the inflection point of the early-RGB-onset class (*C* = 1), β_1_. Finally, in the no-RGB class (*C* = 5), the parameters γ_5_, θ_5_, α_5_, and β_5_ were fixed to zero, and τ_05_ was fixed to −15. Taken together, our basic LCA model estimated only six measurement parameters (Equation 1), and four latent class proportions π_1_ to π_4_ ($\pi_{5}=1-\overset{K-\;1}{\underset{k=1}{\sum }}\pi_{k}$).

The LCA model was extended by the inclusion of covariates predicting class membership. This was accomplished by means of a multinomial logit model so that:

(2) $$P\left(C_{i}=k|x_{i}\right)=\frac{\exp\left(\omega_{0k}+\sum_{j=1}^{J}\omega_{1kj}x_{ij}\right)}{\sum_{l=1}^{l=5}\exp\left(\omega_{0l}+\sum_{j=1}^{J}\omega_{1lj}x_{ij}\right)},$$

with *x*_*i*_ being the individual *i'*s *J* × 1 vector of covariate values with entries *x*_*ij*_ for covariates *j* = 1, 2, …, *J*, and ω parameters standing for multinomial intercepts and weights that were fixed to zero for the no-RGB class *C* = 5. Based on the estimates of the ω-parmameters, RGB probability curves, expected at specific values of the covariate *x*_*i*_, were derived by combining Equations (1, 2) to:

(3) $$\begin{array}{r} P\left(y_{ip}=1|x_{\text{i}}\right)=\overset{K}{\underset{k=1}{\sum }}P\left(C_{i}=k|x_{\text{i}}\right)P\left(y_{ip}=1|C_{i}=k\right). \end{array}$$

Most covariates were observed but, in the case of task enjoyment, we used latent variables that were derived from a linear growth model specified as:

(4) $$\begin{array}{r} z_{ip}=\delta w_{ip}+\eta_{0i}+\frac{p-1}{36-1}\eta_{1i}+\varepsilon_{ip}, \end{array}$$

where *z*_*ip*_ is the individual *i*'s enjoyment score in position *p*, *w*_*ip*_ stands for the values of the item-level covariate as defined before, and δ is a corresponding regression weight. The latent variables η_0*i*_ and η_1*i*_ represent the individual's initial enjoyment value and the rate of change, while ε_*ip*_ is a random disturbance. The η-variables were assumed to follow a bivariate normal distribution. Disturbances were assumed to have zero means, to be normally distributed, and to be uncorrelated with each other as well as with any other variable in the system. The variances of disturbances were set to be equal across positions, but were allowed to be different for text-only and text-picture items. The η-variables were entered into the LCA models similar to *x*-variables (Equation 2), where all growth and LCA parameters were jointly estimated.

All estimations were carried out with the M*plus* 8.0 program (Muthén and Muthén, 2017) using marginal maximum likelihood estimation. Parameter estimates were accompanied by robust standard errors adjusted for non-normality. As LCA models are known to be prone to local minima, we used multiple random start values to check whether the best log-likelihood could be replicated. Model-data fit was evaluated by information theoretic indices including the Akaike information criterion (AIC), Bayesian information criterion (BIC), and the sample size-adjusted BIC (sBIC). These indices take model complexity (i.e., the number of parameters) into account and penalize highly parametrized models.

In order to test whether variables were associated with RGB, we performed multivariate Wald tests of multinomial logit regression weights (Equation 2). The first test served as a test of no association (NA), in which we simultaneously tested all weights attached to a covariate *x*_*j*_ against zero (i.e., ω_11*j*_ = ω_12*j*_ = ω_13*j*_ = ω_41*j*_ = 0). The second test was a test of constant associations (CA) and examined the equality of logistic regression weights (i.e., ω_11*j*_ = ω_12*j*_ = ω_13*j*_ = ω_41*j*_). The CA test is interesting because it indicates whether the effects of covariates on RGB differ between regions (i.e., item positions) in the test. For example, if a covariate is significantly related to RGB (i.e., significant NA test), but the covariate's effects do not differ from each other (i.e., non-significant CA test), it implies that the covariate's effects on RGB constantly increase across item positions (i.e., the curves expected for two values of the covariates have similar shapes but different gradients). In contrast, a significant CA test indicates that the effects of a covariate do not constantly increase across positions, which means that the probability curves predicted at different values of the covariate differ in their shapes. For example, it might turn out that the effect of a covariate is limited to the first latent class (*C* = 1), whereas its effects on classes *C* = 2 and *C* = 3 are near to zero. Imagining this case, differences in RGB probabilities at different levels of the covariate would already arise early in the test session and would then remain constant across subsequent item positions. Alternatively, if the covariate's effects turn out to be stronger on class *C* = 3 and close to zero on classes *C* = 2 and *C* = 1, it means that the covariate's effects emerge only in the last section of the test. Hence, the CA test does not indicate a certain type of relationship. Instead, it indicates a non-constant pattern of relationships.

## Results

### Unconditional LCA Models

**Figure 1:**
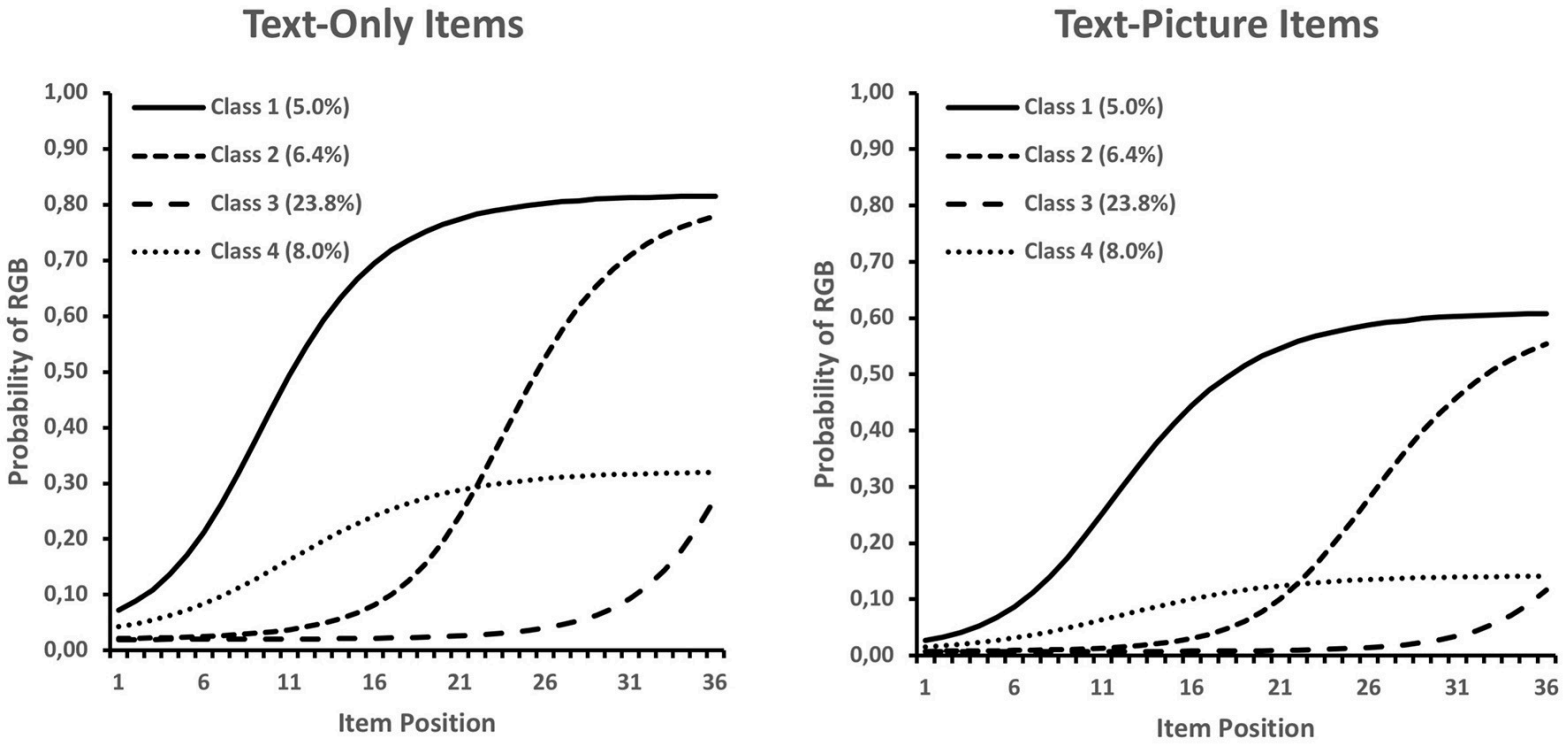
RGB probability for latent Class 1 (early onset point), Class 2 (intermediate onset point), Class 3 (late onset point), and Class 4 (constantly low RGB) with results for text-only (left) and text-picture items (right).

**Figure 2:**
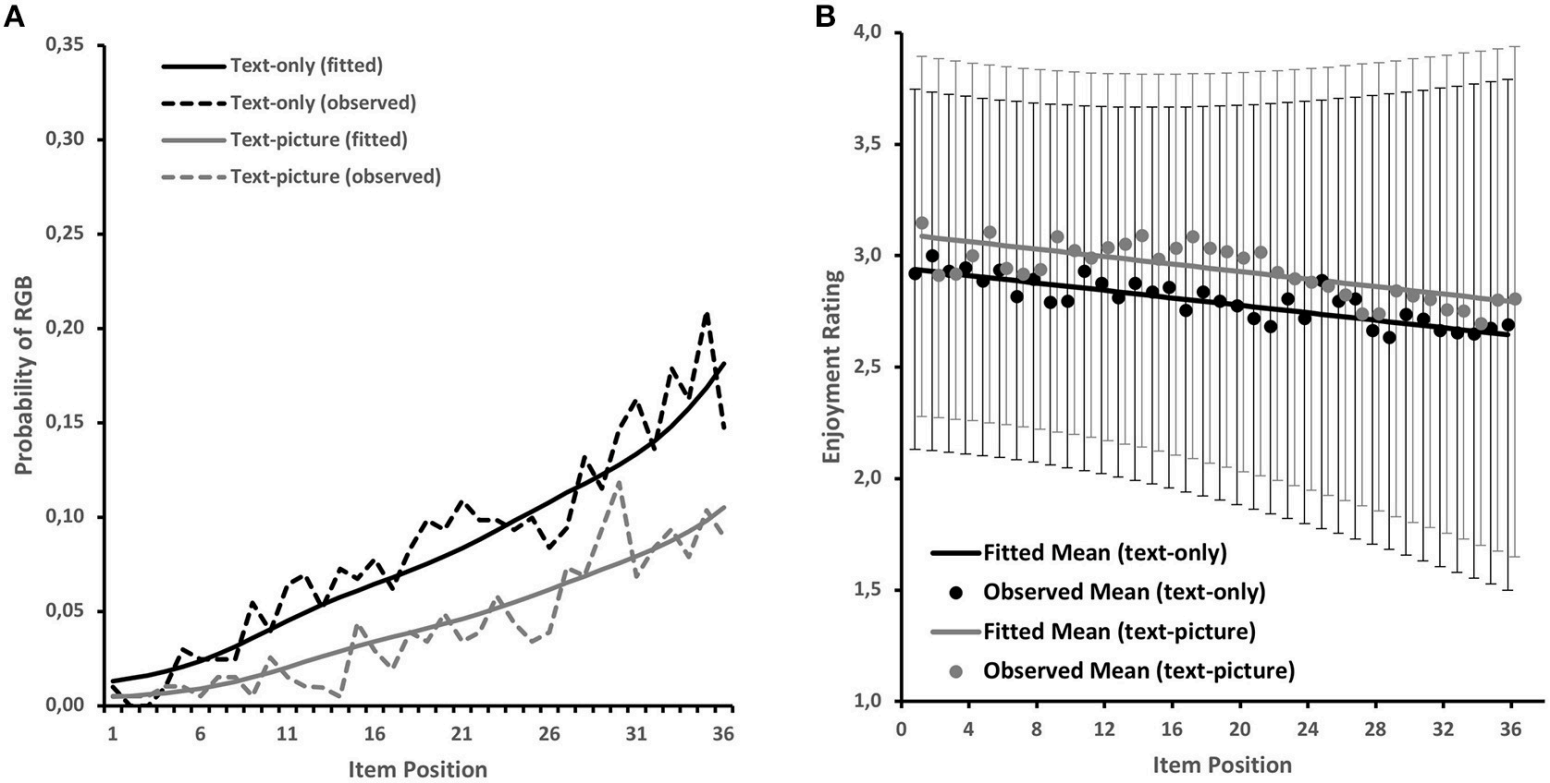
**(A)** Observed and model-fitted RGB probabilities for text-only and text-picture items. **(B)** Observed (dots) and fitted (lines) average enjoyment ratings across item positions and distribution of fitted ratings (10th−90th percentiles) for text-only and text-picture items.

In a first step we employed LCA models that did not include any covariates. The analyses served mainly descriptive purposes and were further used to evaluate the model's ability to depict the marginal RGB probabilities. Our proposed LCA model fitted the data better than a comparison model that assumed two classes (students with no or some RGB) in which the thresholds of all RGB indicators were unconstrained in the RGB class and estimated differently for text-only and text-picture items (unconstrained two-class model: #Parameters = 71, Log Likelihood = −2,313.5, AIC = 4,769.1, BIC = 5,052.3, sBIC = 4827.0; present model: #Parameters = 10, Log Likelihood = −1,963.1, AIC = 3,946.2, BIC = 3,986.0, sBIC = 3,954.3). This result indicates that our LCA model provided a good description of RGB. Figure 1 presents the class-specific RGB probabilities by item position, uncovered by our LCA model, whereas the model fitted and observed RGB proportions are presented in the first panel of Figure 2. In line with previous results, the LCA model indicated that text-only items were more strongly affected by RGB ($\hat{\gamma }$ = −1.05, *SE* = 0.17, *p* < 0.001). Furthermore, the LCA model categorized 56.6% of respondents as not engaging in RGB (observed data: 63.9%).

With respect to the onset of RGB, the LCA indicated that most students started to switch to this behavior in the later part of the test (23.8% in Class 3). The remaining classes had quite similar proportions, ranging between 5.0 and 8.0% (Figure 1). As can be seen in Figure 2, the five classes were sufficient for describing the marginal distribution of RGB for both text-only and text-picture items. Hence, the model appeared to be a solid starting point for assessing the predictors of RGB.

Next, we investigated changes in students' enjoyment ratings over the course of the test. We started with a linear growth curve model that was fitted to the data without considering the remaining variables. The model indicated that text-picture items were associated with higher enjoyment ratings throughout the test-taking session ($\hat{\delta }$ = 0.15, *SE* = 0.02, *p* < 0.001), and that enjoyment ratings were, on average, high at the beginning of the test (${\hat{\mu }}_{\eta_{0}}$ = 2.94, *SE* = 0.04, *p* < 0.001) but decreased on average across positions (${\hat{\mu }}_{\eta_{1}}$ = −0.29, *SE* = 0.04, *p* < 0.001). The results provide evidence for the existence of individual differences in initial enjoyment levels (${\hat{\sigma }}_{\eta_{0}}^{2}$ = 0.39, *SE* = 0.03, *p* < 0.001) and changes in enjoyment (${\hat{\sigma }}_{\eta_{1}}^{2}$ = 0.52, *SE* = 0.05, *p* < 0.001), with the two components being only weakly related (${\hat{\rho }}_{{\eta_{0},\eta }_{1}}$ = −0.12, *SE* = 0.06, *p* = 0.049). Hence, the growth curve model indicated that, regardless of their initial enjoyment level, students exhibited relatively large individual differences in enjoyment declines. This aspect is visualized in Figure 2B, where the model-predicted average declines are depicted together with the observed means and the distribution of model-predicted scores (10th−90th percentiles of the distribution) that document increasing individual differences in enjoyment due to individual differences in the trajectories.

### Conditional LCA Models

To study the correlates of RGB, we started by employing conditional LCA models in which we used each predictor in isolation without considering the remaining covariates. The exceptions were the two latent variables of the growth curve model applied to the enjoyment variables that were investigated simultaneously. Table 1 presents multinomial regression weights determined for each variable and the corresponding tests for no association (row NA) and constant associations (row CA) with RGB.

**Table 1:** Multinomial logistic regression weights determined separately for each covariate, and corresponding Wald-χ^2^ tests of no association (*NA*) and of constant associations (*CA*).

	**Gender**	**School type**	**General cognitive abilities**	**Working memory capacity**	**Initial task enjoyment**	**Change in task enjoyment**
	**Est. (SE)**	**Est. (SE)**	**Est. (SE)**	**Est. (SE)**	**Est. (SE)**	**Est. (SE)**
C = 1	−0.70(0.51)	−4.25(1.06)^**^	−1.02(0.20)^**^	−2.11(0.40)^**^	−0.84(0.52)	−0.91(0.40)^*^
C = 2	−0.75(0.50)	−10.57(1.30)^**^	−0.80(0.22)^**^	−0.45(0.33)	−0.88(0.36)^*^	−1.27(0.40)^**^
C = 3	−0.32(0.32)	−1.23(0.33)^**^	−0.13(0.21)	−0.30(0.20)	−0.20(0.30)	−0.37(0.34)
C = 4	−0.81(0.50)	−3.03(0.91)^**^	−1.11(0.24)^**^	−1.45(0.39)^**^	−0.72(0.35)^*^	0.13(0.34)
	**χ^2^** **(df)**	**χ^2^** **(df)**	**χ^2^** **(df)**	**χ^2^** **(df)**	**χ^2^** **(df)**	**χ^2^** **(df)**
NA	7.05(4)	113.20(4)^**^	44.37(4)^**^	31.13(4)^**^	11.42(4)^*^	16.07(4)^**^
CA	1.32(3)	59.30(3)^**^	20.00(3)^**^	18.18(3)^**^	3.61(3)	10.94(3)^*^

*Gender: 0 = male, 1 = female; school type: 0 = academic track (Gymnasium), 1 = non-academic track (i.e., regional school); Measures of general cognitive abilities (KFT) and working-memory were standardized prior to the analysis*.

* p ≤ 0.05;

** *p ≤ 0.01*.

As can be seen in Table 1, almost all variables were significantly related to RGB. The exception was gender. The pattern of gender differences was in line with previous results but did not reach the significance threshold (*p* = 0.113). Judged on the value of the Wald-χ^2^ statistic, school type was most strongly related to RGB, whereas the initial level of and change in enjoyment had the weakest relationships to RGB. Furthermore, the four multinomial logistic regression weights belonging to each variable appeared to differ from each other. For example, the regression weights associated with school type indicated that the chances of academic-track students belonging to classes *C* = 1, 2, or 4 vs. class *C* = 5 were much smaller than the corresponding chances of non-academic-track students. In contrast, school-type differences in the relative chance of belonging to class *C* = 3 (i.e., the late RGB onset class) were less pronounced (i.e., the regression weight was closer to zero).

As can be seen in the CA row in Table 1, school type was differentially related to the onset point of RGB, whereas gender and initial enjoyment were not. Academic-track students were least likely to have an early RGB onset (i.e., membership in classes *C* = 1, 2, or 4). Similar relationships were found with the continuous covariates, general cognitive abilities, working-memory capacity, and change in enjoyment, so that students with higher scores on these variables were least likely to have an early RGB onset.

**Figure 3:**
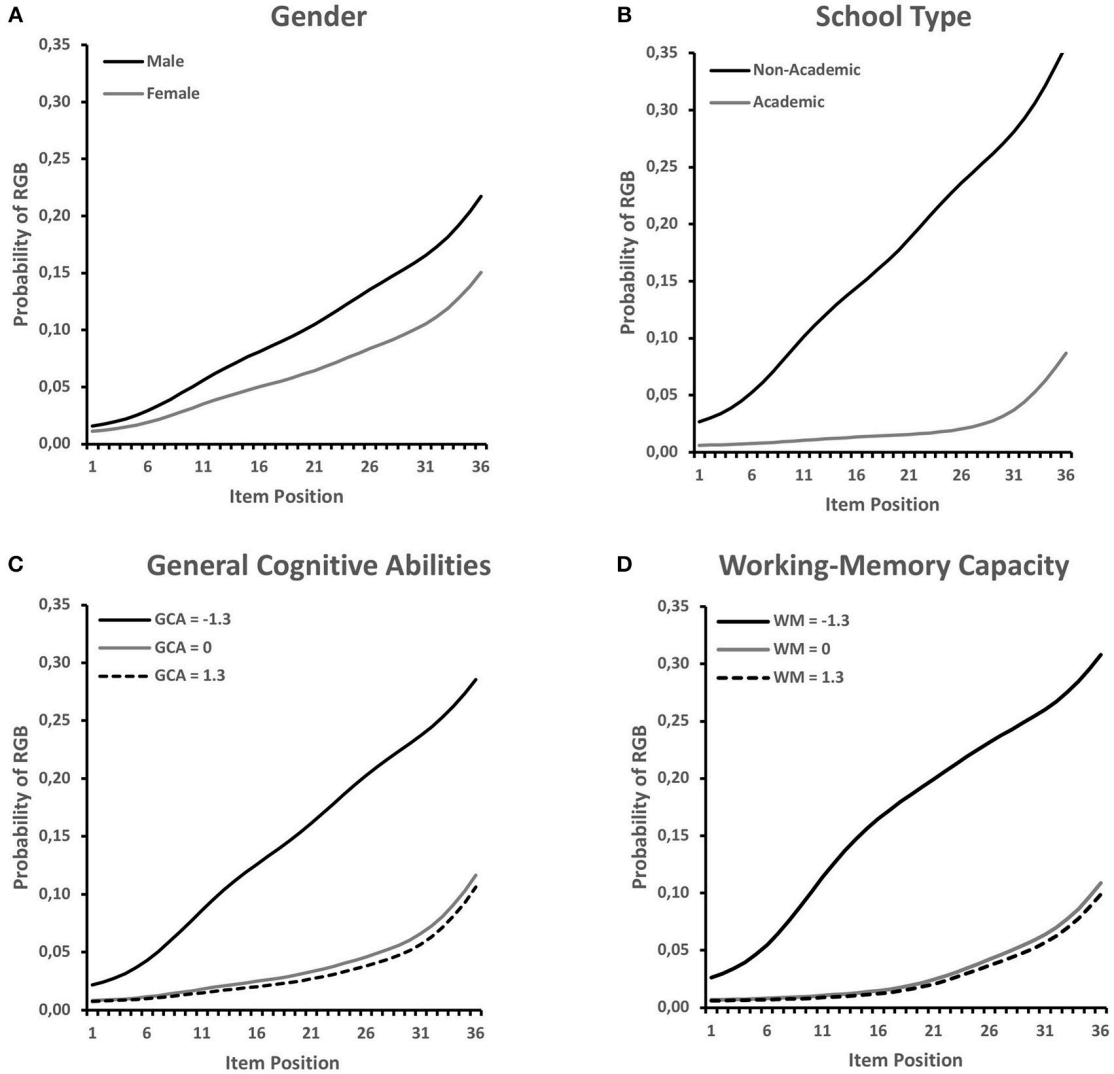
Estimated RGB probabilities by item position expected for different levels of the covariates **(A)** gender, **(B)** school type, **(C)** general cognitive abilities, and **(D)** working-memory capacity. Values ±1.3 standard deviations around the mean were chosen for general cognitive abilities and working-memory capacity because these roughly indicate the 10th and 90th percentiles of their distribution.

In order to get an impression of the pattern of relationships, the model-predicted probabilities of RGB at selected values of the covariates are plotted in Figure 3. As suggested by the non-significant overall effect (NA test, Table 1) and the non-significant CA test, gender differences were rather small, but showed a relatively constant (albeit non-significant, *p* = 0.113) increase across positions. In contrast, differences between school types were clearly larger and showed a strong increase across item positions, whereby the increase was largest in the first two thirds of the test. A similar picture was revealed for the continuous measures of general cognitive abilities and working-memory capacity. In the case of these variables, it appeared that above average scores did not have a meaningful effect on RGB. Rather, students who scored well below average on these tests had a higher probability of engaging in RGB.

**Figure 4:**
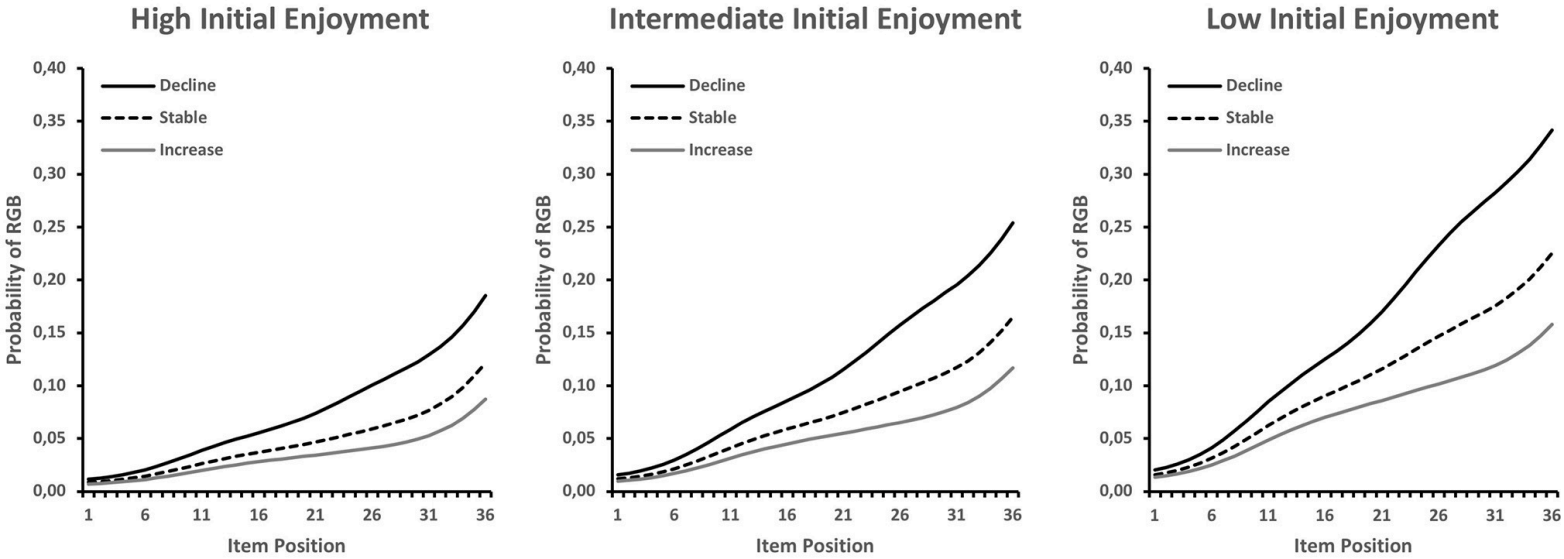
RGB probabilities for text-only items by item position, expected for different combinations of initial enjoyment and change in enjoyment over the course of the test (i.e., item positions).

The relationship of RGB with the repeatedly measured enjoyment variable is shown in Figure 4. In order to account for the initial level and the change component in the enjoyment ratings, the figure contains three line plots for low (10th percentile), average, and high levels (90th percentile) of initial enjoyment, which each contain RGB probability curves for low (10th percentile), average, and high levels (90th percentile) of change in enjoyment. As shown in Figure 4, lower initial levels of enjoyment were associated with constantly increasing levels of RGB across positions (non-significant CA test). As further shown in Figure 4, the RGB probability curves differed at each level of initial enjoyment, depending on the change in enjoyment, so that steeper decreases in enjoyment were associated with steeper increases in RGB (see also NA row in Table 1).

All results presented up to this point pertain to the models in which each covariate was investigated in isolation. However, the majority of student characteristics employed were correlated among each other, as can be taken from Table 2. Even though the correlations were not so high that they could cause collinearity problems, the question about each variable's unique contribution to the prediction of RGB emerged. We approached this question by using all covariates simultaneously as predictors of latent class membership. The results are presented in Table 3.

**Table 2:** Predictor correlations.

	**1**	**2**	**3**	**4**	**5**	**6**
1. Gender	1					
2. School type	−0.039	1				
3. General cognitive abilities	−0.002	0.363^**^	1			
4. Working memory	0.025	0.298^**^	0.281^**^	1		
5. Initial task enjoyment	0.024	0.066	0.060	−0.028	1	
6. Change in task enjoyment	0.086	0.076	0.099	0.039	−0.123^*^	1

* p ≤ 0.05;

** *p ≤ 0.01*.

**Table 3:** Multinomial logistic regression weights determined jointly for all covariates, and corresponding Wald-χ^2^ tests of no association (*NA*) and of constant associations (*CA*).

	**Gender**	**School type**	**General cognitive abilities**	**Working-memory capacity**	**Initial task enjoyment**	**Change in task enjoyment**
	**Est. (SE)**	**Est. (SE)**	**Est. (SE)**	**Est. (SE)**	**Est. (SE)**	**Est. (SE)**
C = 1	−0.84(0.79)	−4.77(0.97)^**^	−0.60(0.36)	−2.54(0.69)^**^	−1.39(0.67)^*^	−1.22(0.68)
C = 2	−0.86(0.75)	−3.30(0.37)^**^	−0.38(0.21)	−0.26(0.42)	−1.18(0.49)^*^	−1.12(0.50)^*^
C = 3	−0.59(0.33)	−1.37(0.43)^**^	0.13(0.20)	−0.22(0.20)	−0.34(0.32)	−0.29(0.32)
C = 4	−1.07(0.69)	−3.00(0.77)^**^	−0.80(0.30)^**^	−1.24(0.49)^**^	−1.10(0.56)	0.02(0.47)
	**χ^2^** **(df)**	**χ^2^** **(df)**	**χ^2^** **(df)**	**χ^2^** **(df)**	**χ^2^** **(df)**	**χ^2^** **(df)**
NA	6.14(4)	99.94(4)^**^	9.59(4)^*^	17.69(4)^**^	11.52(4)^*^	10.12(4)^*^
CA	0.68(3)	33.85(3)^**^	9.15(3)^*^	12.07(3)^**^	5.27(3)	7.63(3)

*Gender: 0 = male, 1 = female; school type: 0 = academic track (Gymnasium), 1 = non-academic track (i.e., regional school); Measures of general cognitive abilities (KFT) and working-memory were standardized prior to the analysis*.

* p ≤ 0.05;

** *p ≤ 0.01*.

The (non-significant) relationship of gender with RGB was not affected by the inclusion of the other covariates (see Table 1). A similar result was found for school type; RGB was still significantly related to this variable and also strongly related to an early RGB onset. The relationship of general cognitive abilities with RGB was clearly reduced after all covariates were included in the model, although the relationship with RGB and RGB onset remained significant. In contrast, the relationship of working memory with RGB was similar to that of the previous model (see Table 1), which means that it continued to be significantly related to RGB and its onset. Initial enjoyment also remained significantly related to RGB, but the regression weights for the different latent classes did not differ significantly (CA test; see Table 3). Finally, changes in enjoyment continued to be significantly related to RGB, but the CA test was no longer significant on the *p* < 0.05 level (*p* = 0.054). This weakens the evidence of a strong relation between students' enjoyment decline and early RGB onset.

## Discussion

The present study examined the correlates of RGB onset and its temporal dynamic over the course of testing as a between-student factor with regard to motivational and cognitive student characteristics, using a latent class approach as a base for our analyses. Specifically, we investigated the extent to which different patterns of (early) RGB onset were related to cognitive and motivational covariates in order to gain deeper insights into the processes that may underlie disengaged test-taking behavior in low-stakes assessment. In the following sections, we discuss the key results of the study with regard to our hypotheses, the theoretical assumptions, and earlier research. Finally, we reflect on the study's limitations, consider future research suggestions, and close the article with an overall conclusion and a consideration of the practical significance of our findings.

### Student Characteristics

Testing our hypothesis regarding the relation of RGB or RGB onset and students' gender (H1a), we did not find a significant relation, contrary to our expectation. However, this is not entirely surprising, as the findings in the literature are also inconsistent. Several studies indicate that male students have lower levels of test-taking motivation and also tend to show disengaged behavior, such as RGB, more often (for a review see e.g., DeMars et al., 2013). Still, not all studies find a significant relation between gender and RGB (e.g., Wise et al., 2009). In the present study, as can be seen in Figure 3A, the descriptive pattern was in line with the expectation that male students would engage in RGB earlier than female students, but the coefficient did not reach significance. This result seems to be primarily related to a power issue, as the present sample may not have been large enough to significantly show the effect. Generally, the relationship between RGB and gender appeared to be of lower practical importance considering the marginal effect sizes in vast representative samples, such as in the study by Goldhammer et al. (2016). However, gender differences in RGB may be more pronounced in younger students, which seemed to be reflected at a descriptive level in our data. The moderating role of students' age would, thus, be an interesting factor for future research.

Confirming our hypothesis regarding students' school-type attendance (H1b), we found a remarkably higher risk of an earlier RGB onset and a stronger increase of RGB probabilities in students from non-academic-track schools (see Figure 3B). This effect remained significant when all predictors were included in one model; moreover, school type was the strongest predictor of early RGB onset. In the German school system, which assigns students to different secondary school tracks based on their performance in elementary school, school type is strongly related to students' academic abilities (e.g., Prenzel et al., 2013). In addition, school type has been shown to be connected to differences in students' motivation to work in an effortful way in low-stakes assessments (e.g., Baumert and Demmrich, 2001; Nagy et al., 2018b). Thus, both factors, academic ability and motivation, are probably reflected in the substantial RGB differences between school tracks. Earlier studies have shown similar relations of RGB (e.g., Lee and Jia, 2014; Goldhammer et al., 2016; Wise and Gao, 2017) or item position effects (e.g., Nagy et al., 2016, 2018a) with students' academic ability level (e.g., SAT scores; Wise et al., 2009) or school-type attendance (Nagy et al., 2016). Nevertheless, some studies did not find ability-related differences in students' response effort (e.g., Wise and DeMars, 2005; Wise and Kong, 2005; Wise et al., 2006a). These mixed results might be attributed to the different sample characteristics, test situations, and criteria used to judge students' academic ability (e.g., scores from the investigated test vs. external criteria, such as SAT scores). In this study, we used a criterion that is independent of students' test achievement and known to be a solid indicator of academic abilities. However, while the investigated data set included students from academic- and non-academic-track schools, it did not reflect the full width of German non-academic-track schools (i.e., no lower secondary schools). Our findings might therefore not fully represent school-type differences, as students from lower non-academic schools might further contribute to the unfavorable picture of school-type differences in RGB.

In line with our hypotheses regarding students' general cognitive abilities (H1c) and working-memory capacity (H1d), we found substantial evidence that both factors are significantly related to RGB and predict an earlier RGB onset and a stronger increase in RGB. However, this only applied to students with relatively low cognitive capacities (see Figures 3C,D). This indicates that a lack of cognitive resources raises students' risk of engaging in RGB early on and of showing a stronger RGB increase. Building on expectancy-value models (e.g., Eccles and Wigfield, 2002) and the DCM assumptions (Wise, 2017), this is not really surprising. However, so far, we are not aware of any empirical studies that have investigated standardized cognitive ability tests as predictors of RGB development so far. While both of our measures were clearly related to RGB as isolated predictors, it is especially interesting that working-memory capacity seemed to be more predictive of both RGB and RGB onset than general cognitive abilities. This became evident when we integrated all indicators into one full competitive model, where the general cognitive ability covariate lost a substantial part of its explanatory power but the working-memory factor remained basically unaffected. This might be explained as follows: Whereas general cognitive abilities are assumed to be more or less stable across situations and time (i.e., fluid intelligence as a trait), working-memory capacity is known to be subject to stronger situational fluctuations (see e.g., Hofmann et al., 2011) and can also be subject to mental fatigue effects that undermine attentional control (Schmeichel, 2007). However, executive attention is a key factor in self-controlled behavior, which is also needed in any test situation in order for students to focus on the posed problems and to solve them with effort. This demand tends to become aversive over the course of testing time (Inzlicht et al., 2014). This relation could help to explain why working-memory capacity seems to be the more important cognitive resource required for engaged test-taking behavior over the course of a test session.

### Task Enjoyment Over the Course of Testing

RGB is typically interpreted as an indicator of student motivation. In our study, we examined the extent to which RGB was related to students' perceived motivation level as an open question. By modeling the intercept of students' multiple enjoyment ratings across the test session as a latent covariate in the LCA, we tested Hypothesis H2a. Although there was evidence for a relation between students' initial enjoyment (i.e., rating of the first item) and RGB, we did not find a significant relation to RGB onset (Figure 4). This was true for both the isolated analysis of initial enjoyment as a single predictor and the full model with all predictors. The observed and model-fitted data of students' enjoyment ratings (Figure 2B) showed a decrease over the course of the test session, as expected, though the mean level of students' enjoyment remained relatively high. The figure also shows that there was a lot of inter-individual variance; we investigated this variance by integrating students' estimated slopes as a latent covariate into our LCA to test Hypothesis H2b. This provided tentative evidence that a negative enjoyment trajectory over time predicted both RGB and RGB onset in the isolated model. However, the relation with RGB onset did not remain significant when competitive covariates were added to the model, which weakens the evidence for Hypothesis H2b to some extent.

Overall, students' enjoyment ratings were not strongly related to their RGB tendency when compared to the cognitive covariates. This relatively weak relation could be due to the young age of the students in the current sample, who might not yet be able to correctly reflect on their current enjoyment; but, it could also indicate that test-takers simply have problems with an accurate evaluation of their motivational state. However, this question cannot be answered based on the present findings. Penk and Richter (2017) recently applied a comparable approach of modeling ninth-graders' test-taking motivation across a test session to investigate item position effects. They found that initial test-taking motivation was a better predictor of the item position effect than changes in motivation. This pattern is the opposite of our results and is somewhat surprising; it indicates that there are interesting questions to be answered in future research on test-taking motivation.

### Limitations and Future Directions

Some limitations need to be taken into account when interpreting the present findings. First, the current sample cannot be considered representative, which constrains the generalizability. The effects of school type might be biased because we did not include all German school tracks and we tested only students in the fifth and sixth grades. Compared to typical large-scale assessments, the current sample was rather small but seemed to be sufficient, except for determining the relation between RGB and gender, which may have been underpowered. As an unusual advantage, however, the data included important measures, such as the repeated enjoyment rating and the indicators of students' general cognitive abilities and working-memory capacity, which were at the core of the present analyses. The test circumstances were highly comparable to typical computer-based low-stakes testing programs. Nevertheless, future studies should challenge our research and try to replicate the current findings in larger data sets. Especially a transfer of our latent class approach to other samples would be desirable to evaluate the extent to which the presumptions and findings of our study (e.g., the proportion of student assignments to the five individual LCA classes) are robust. As such, the proposed analysis could be a fruitful base for future research on the determinants of RGB onset and its dynamics across testing time.

Second, the reliability of our working-memory test (i.e., reversed digit span) was, unfortunately, not very high (α = 0.65). However, a tradeoff has to be made with view to the challenge of measuring working-memory indicators in group sessions, as individual test sessions can better ensure that the test is administered in the best way possible. It would therefore be advantageous to reexamine the current issue by assessing other or additional working-memory capacity indicators that have a higher test reliability.

A third potential limitation pertains to the fact that both the science test and the cognitive tests (KFT N2 and reverse digit span) were administered in the same test session. The results might therefore share common variance due to a general tendency of students to work seriously on test items in a low-stakes situation (i.e., in terms of a latent trait) and also due to their current overall compliance with the test-taking situation (i.e., in terms of a current state during the specific test administration). However, the cognitive tests were presented before the science test. The risk that students' behavior was already effortless at the beginning of the test session is rather low. This assumption is supported by the observation that only a small number of RGB trials occurred in the first items of the science test, indicating that most students were still prepared to make an effort to work on the test items at the beginning of the test. Nevertheless, test scores from standardized cognitive tests that were assessed in different sessions from another day would have been preferable.

### Conclusion and Implications for Educational Practice

Drawing on a theory-driven latent class model, standardized measures of students' cognitive abilities, and repeated ratings of their current item-solving enjoyment, this study was able to extend previous work and widen the understanding of RGB. The main strength of our investigation is that our LCA approach made it possible to study the dynamics of RGB in connection with several indicators of cognitive and motivational resources at a student level. In brief, we found evidence that students' item-solving enjoyment, academic ability, and cognitive capacities are (closely) related to the RGB onset point and the dynamics of RGB across a low-stakes test session. Students from non-academic-track schools, students with low general cognitive abilities and low working-memory capacity, as well as students with a stronger decline in their task enjoyment over the course of the test were substantially more likely to engage in RGB earlier in the test and to progress with that behavior. All of these findings are in line with the theoretical assumptions from expectancy-value models (e.g., Eccles and Wigfield, 2002) as well as those of the DCM by Wise and Smith (2011). However, future research should also focus on non-cognitive factors, such as coping strategies, text anxiety or the well-being of students and on the relations of these factors to test-taking behavior. In addition, characteristics of students' home environment, such as the socio-economic status of their parents and school culture, including the school climate, the ethnic composition and the value teachers, parents and peers attribute to learning and testing efforts, should be taken into account in order to better understand RGB from a broader perspective.

Alongside the new support they provide for the theoretical models concerning the psychological determinants of RGB, our results also have practical implications. The substantial relation of RGB to students' academic and cognitive abilities suggests that students' test engagement seems to be a seriously, confounding factor (in terms of true competences) for a valid interpretation of school-type comparisons of low-stakes test performances (see also Wise et al., 2009; Nagy et al., 2018b). This is a problem because such comparisons are often an important goal of large-scale testing programs. Furthermore, all motivation-filtering procedures rely on the theoretical assumption that student motivation is unrelated to true proficiency. However, if this criterion is not fulfilled, the filtering procedure induces bias. In particular, filtering students with low proficiency out of the data would provide an overly positive picture of the performance in the investigated sample, leading to an overestimation of true proficiency. In addition to the attempt of using statistical correction procedures, this problem should also be discussed at the level of test characteristics. For example, applying shorter tests, using items with a more appealing design (see e.g., Lindner et al., 2017b, Wise et al., 2009), and possibly having longer breaks between different test blocks may foster students' test-taking motivation and could allow them to refresh exhausted cognitive resources before continuing to focus their attention on further tasks (see also Lindner et al., 2018). In the light of the current results, such considerations seem to be particularly relevant for students from non-academic-track schools and for students with low working-memory capacity. However, the extent to which an improvement in assessment conditions would actually contribute to solving the problems that are connected to low test effort is a question for future research.

## Ethics Statement

This study was carried out in accordance with the Declaration of Helsinki and the ethical guidelines for experimental research with human participants as proposed by the German Psychological Society (DGPs). Prior to the test session, we obtained written informed consent from all students and their legal guardians.

## Author Contributions

All authors have made a substantial intellectual contribution to the article and approved it for publication. ML developed the study design, performed the data collection, prepared the data for analyses and wrote the manuscript. GN developed the conception of the analyses, performed the analyses and co-wrote the statistical analyses paragraph. GN and OL reviewed andedited the article. All authors contributed to the theoretical conception, the interpretation of the results and manuscript revisions.

### Conflict of Interest Statement

The authors declare that the research was conducted in the absence of any commercial or financial relationships that could be construed as a potential conflict of interest.

## Footnotes

^1^Wise and Gao (2017) recently proposed a broader measure of test-taking effort, which they refer to as response behavior effort (RBE) and response behavior fidelity (RBF), which makes it possible to identify rapid omits and rapid perfunctory answers to constructed response items in addition to RGB.
